# A theory of decision-making using diffusion-to-bound models: choice, reaction-time and confidence

**DOI:** 10.1186/1471-2202-15-S1-P88

**Published:** 2014-07-21

**Authors:** Philipp Schustek, Rubén Moreno-Bote

**Affiliations:** 1 Research Unit, Parc Sanitari Sant Joan de Deu and Universitat de Barcelona, Esplugues de Llobregat, Barcelona, Spain; 2 Centro de Investigación Biomédica en Red de Salud Mental (CIBERSAM), Esplugues de Llobregat, Barcelona, Spain

## Introduction

Diffusion-to-bound models are typically found to be good models for many aspects of perceptual decision-making tasks, and they describe with high level of accuracy both the psychometric and chronometric curves of humans and non-humans. In these models, the decision process is viewed as a state variable that evolves randomly over time and represents the noisy evidence accumulated so far, and a decision is formed when this diffusing state variable hits one of two decision boundaries. However, and somehow surprisingly, diffusion-to-bound models are at the same time considered to be problematic models for the description of decision confidence in decision-making tasks [1,2]. In this abstract we show that this view is not accurate.

## Results

We found explicit analytical expressions for decision confidence in diffusion-to-bound models as a function of all relevant parameters of the stimulus and task, such as threshold height, stimulus discriminability and stimulus noise [3,4]. Using these analytical results we show that, contrary to the common wisdom, diffusion-to-bound models are able to reproduce all known effects of decisions confidence: 1) decision confidence decreases with reaction time in reaction time tasks, 2) confidence increases with time in fixed-time tasks, 3) decision confidence is lower in speed compared to accurate conditions, 4) confidence increases with stimulus discriminability, 5) confidence is higher than actual performance for easy conditions and lower than actual performance for difficult conditions (the so-called “hard-easy” effect), 6) confidence is higher in correct than in incorrect trials (the so-called “resolution of confidence”), and 7) confidence is increasingly resolved with time pressure. Furthermore, our theory also predicts quantitatively the way decision confidence depends on stimulus and tasks parameters, such as stimulus discriminability, range of stimuli and speed-accuracy conditions, and on decision parameters, such as reaction time. We note, however, that current estimation procedures of confidence might be biased highly because confidence is often verbally self-reported, and stress the necessity of more objective measurements of decision confidence.

## Conclusions

We have found analytical expression for decision confidence in diffusion-to-bound models and showed that this expression fully accounts for all known effects of decision confidence. Furthermore, our model makes quantitative predictions that could be tested experimentally.
